# Leg pain in neuropathic postural tachycardia syndrome is associated with altered muscle membrane properties

**DOI:** 10.1007/s10286-021-00830-5

**Published:** 2021-10-21

**Authors:** Belén Rodriguez, Karin Jost, Lotte Hardbo Larsen, Hatice Tankisi, Werner J. Z’Graggen

**Affiliations:** 1 Department of Neurosurgery, Inselspital, Bern University Hospital, Bern, Switzerland; 2 Department of Neurology, Inselspital, Bern University Hospital, Bern, Switzerland; 3 Department of Clinical Neurophysiology, Aarhus University Hospital, Aarhus, Denmark

**Keywords:** Muscle velocity recovery cycles, Ischemia, Autonomic dysfunction, Acrocyanosis, Venous pooling, Tilt table testing

## Abstract

**Purpose:**

In neuropathic postural tachycardia syndrome, peripheral sympathetic dysfunction leads to excessive venous blood pooling during orthostasis. Up to 84% of patients report leg pain and weakness in the upright position. To explore possible pathophysiological processes underlying these symptoms, the present study examined muscle excitability depending on body position in patients with neuropathic postural tachycardia syndrome and healthy subjects.

**Methods:**

In ten patients with neuropathic postural tachycardia syndrome and ten healthy subjects, muscle excitability measurements were performed repeatedly: in the supine position, during 10 min of head-up tilt and during 6 min thereafter. Additionally, lower leg circumference was measured and subjective leg pain levels were assessed.

**Results:**

In patients with neuropathic postural tachycardia syndrome, muscle excitability was increased in the supine position, decreased progressively during tilt, continued to decrease after being returned to the supine position, and did not completely recover to baseline values after 6 min of supine rest. The reduction in muscle excitability during tilt was paralleled by an increase in lower leg circumference as well as leg pain levels. No such changes were observed in healthy subjects.

**Conclusions:**

This study provides evidence for the occurrence of orthostatic changes in muscle excitability in patients with neuropathic postural tachycardia syndrome and that these may be associated with inadequate perfusion of the lower extremities. Insufficient perfusion as a consequence of blood stasis may cause misery perfusion of the muscles, which could explain the occurrence of orthostatic leg pain in neuropathic postural tachycardia syndrome.

## Background

**Fig. 1:**
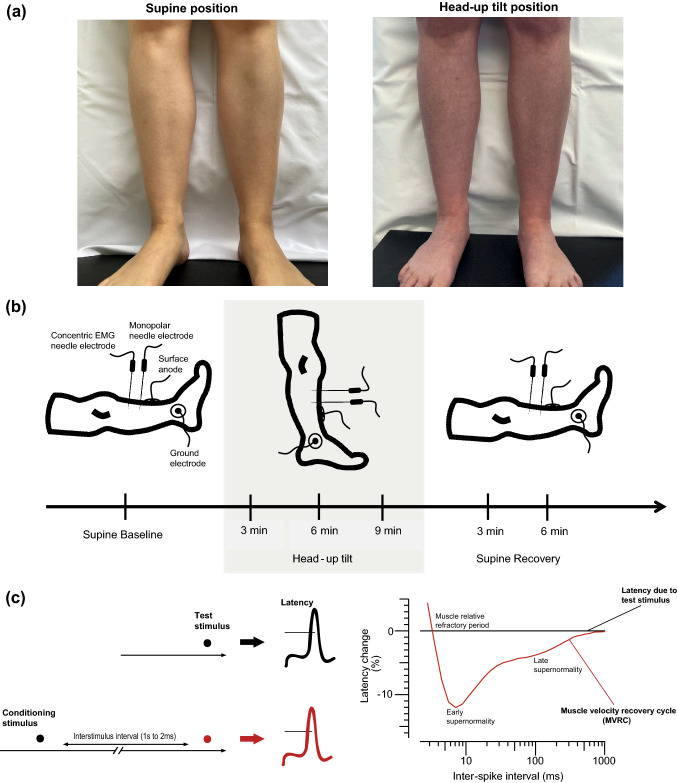
Technique of recording muscle velocity recovery cycles and illustration of the study protocol. **a** Legs of a patient with postural tachycardia syndrome, on the left side in the supine position and on the right side after 10 min of head-up tilt, showing acrocyanosis during head-up tilt. **b** Illustration of electrode arrangement for recoding muscle velocity recovery cycles and of the study protocol. A monopolar needle electrode (cathode) was inserted into the distal third of the tibialis anterior muscle. A surface electrode (anode) was placed further distally. Recordings were made with a concentric electromyography (EMG) needle that was placed proximal to the stimulating needle along the course of the muscle fibres. The surface ground electrode was placed above the malleolus lateralis. Six muscle velocity recovery cycles were recorded: one in the supine position before head-up tilt, one at the beginning, middle and end of 10 min of head-up tilt, and two during 6 min of supine recovery after head-up tilt. **c** On the left side, illustration of the technique of recording multi-fibre muscle velocity recovery cycles. The technique measures latency changes in action potential due to a test stimulus as a consequence of a preceding conditioning stimulus, which is applied at variable interstimulus intervals. On the right side, illustration of a muscle velocity recovery cycle with percentage changes in latency due to a conditioning stimulus, plotted as a function of interstimulus interval

Postural tachycardia syndrome (POTS) is a chronic disorder of the autonomic nervous system. Typical orthostatic symptoms in POTS include light-headedness, palpitations, blurred vision, cognitive dysfunction, generalized weakness, dyspnoea and headache. Many patients also experience non-orthostatic symptoms such as bowel and bladder dysfunction, fatigue, sleep disturbance and exercise intolerance [1, 2]. The aetiology of POTS is heterogeneous, and several pathophysiological processes are thought to be responsible for the occurrence of tachycardia while standing [2, 3]. Different subtypes have been identified, the most common of which is referred to as neuropathic POTS, with an estimated frequency of at least 50%. Neuropathic POTS is characterized by dysfunction of the peripheral sympathetic nerves predominantly affecting the lower limbs. The consequently impaired peripheral vasoconstriction leads to excessive venous blood pooling during orthostasis [2, 4]. Patients with this subtype typically experience position-dependent acrocyanosis in the lower extremities and a red-blue marbled skin that feels cold to the touch (for illustration see Fig. 1a) [2, 5]. Up to 84% of patients report symptoms of the lower extremities such as muscle pain and foot coldness, and 83% complain of muscle weakness [6, 7]. Despite these symptoms being so frequent, their aetiology has not yet been clarified, and consequently current therapy options are limited.

The recording of muscle velocity recovery cycles (MVRCs) is an electrophysiological method that allows us to investigate the function and properties of muscle fibre membranes in vivo [8, 9]. This method is based on the principle that a muscle action potential is followed by two phases of altered muscle membrane excitability. Immediately after an action potential, a phase of reduced excitability occurs (refractory period), which is followed by a second phase of heightened excitability (early and late supernormality). The latter depends on the depolarizing afterpotential, which is an indicator of the charge left on the capacitance of the muscle fibre membrane [8, 10]. MVRCs can be recorded by measuring the changes in conduction velocity of a muscular action potential after a preceding conditioning stimulus at varying interstimulus intervals. This provides an indirect measurement of the afterpotential and allows conclusions to be drawn about the membrane potential and function [8, 11]. For recording of MVRCs, a few muscle fibres are directly stimulated using a monopolar needle electrode (Fig. 1b). Stimulus responses are recorded from the same bundle of muscle fibres with a second needle electrode [11, 12]. This technique enables the determination of the phases of altered muscle membrane conduction: muscle relative refractory period (MRRP), early supernormality (ESN) and late supernormality (LSN) (Fig. 1c). MRRP and ESN are highly dependent on the muscle membrane potential, whereas LSN reflects transverse tubular function [8, 10]. The recording of MVRCs has been applied to investigate muscle excitability and the underlying pathophysiological processes of muscle symptoms in various types of diseases [13–18].

To the best of our knowledge, the aetiology of muscle weakness and pain experienced by patients with neuropathic POTS has not been further investigated. The aim of the present study was to examine possible changes in muscle membrane properties in patients with neuropathic POTS as a function of body position and to compare the results to healthy subjects in order to investigate position-dependent leg symptoms in neuropathic POTS.

## Methods and materials

### Participants

Ten patients with confirmed neuropathic POTS and ten healthy subjects participated in this study. All subjects had to be older than 18 years of age, and patients had to have a positive history of orthostatic leg pain. Exclusion criteria for all participants included pregnancy and breastfeeding, and additionally for control subjects vasovagal syncope in the medical history, intake of vasoactive medication, and venous insufficiency. For the diagnosis of neuropathic POTS, medical history, physical and neurological examination, cardiovascular autonomic function testing, thermoregulatory sweat test and/or quantitative testing of sudomotor axon reflexes, measurement of plasma norepinephrine levels, determination of autoantibodies against G-protein-coupled receptors, and in some cases skin biopsies were considered [1, 19]. The mean increase in heart rate during head-up tilt (HUT) at the time of diagnosis was 44 beats per minute (bpm; range 30–55 bpm). All patients had sudomotor dysfunction. In eight patients, autoantibodies against the alpha-1 adrenergic receptor were present, and in two the skin biopsy was pathological. Participants were allowed to drink and eat prior to the examination. Patients took their medications (fludrocortisone, midodrine, pyridostigmine, metoprolol) as usual except for midodrine, which was stopped 4 h before the examination. One patient was treated with intravenous immunoglobulins. This was decided because the study involved examination with needles, which increases the risk of vasovagal syncope during HUT, and fasting would further increase this risk [20]. All procedures were approved by the local ethics committee (Kantonale Ethikkomission Bern, Switzerland) and conformed to the Declaration of Helsinki and its amendments. Patients and healthy subjects gave their written informed consent to participate in the study.

### Study design and experimental protocol

This case–control study investigated muscle membrane properties as a function of body position. To this end, MVRCs were recorded repeatedly during tilt table testing in patients with neuropathic POTS and healthy control subjects. The study protocol is illustrated in Fig. 1b. Blood pressure and heart rate were continuously monitored throughout the procedure. In total, six MVRCs were recorded from the participants’ left tibialis anterior muscle: one in the supine position before HUT, three during 10 min of HUT, and two during supine recovery after HUT. Additionally, the circumference of the participants' right lower leg and their subjective leg pain levels were assessed before and during 10 min of HUT.

### Cardiovascular autonomic function testing

All participants were placed comfortably in the supine position on a tilt table. During the experimental procedure, beat-to-beat blood pressure and heart rate were measured with the Portapres device (Finapres Medical Systems BV, Arnhem, Netherlands) on the left hand. In addition, intermittent brachial blood pressure and heart rate were measured on the right arm in the supine position and during HUT using an automated Dinamap Pro 100 sphygmomanometer (GE Medical Systems, Tampa, FL, USA). Furthermore, a standard three-lead electrocardiogram was recorded throughout testing. All participants underwent standard screening autonomic function tests in the supine position before HUT. The autonomic function screening included a pressure-controlled Valsalva manoeuvre (40 mmHg for 15 s) in the supine position, the investigation of the pressor response to a cutaneous cold stimulus at the wrist, and assessment of the heart rate variability in response to deep breathing [21]. HUT was performed at a tilt angle of 60°.

### Recording of muscle velocity recovery cycles

#### Stimulation

MVRC recordings were performed from the participant’s tibialis anterior muscle of the left leg as described in detail in prior studies [22, 23]. This muscle was chosen based on its easy accessibility and its clearly defined endplate zone, which prevents stimulation of the motor axons, and because it is one of the muscles in the lower limbs that has been successfully and reliably tested in previous studies [9, 22]. A monopolar insulated needle electrode (25 mm × 26 G, TECA, Natus Manufacturing Limited, Galway, Ireland) served as cathode through which the stimulation currents were delivered. This needle was perpendicularly inserted into the distal third of the tibialis anterior. A non-polarizable surface electrode (Red Dot, 3 M Health Care, Borken, Germany) was used as anode and was placed distal to the cathode (Fig. 1b). For stimulation, stimulus waveforms (rectangular current pulses of 0.05 ms duration) were generated by a computer and converted to current with an isolated constant-current stimulator (DS5, Digitimer Ltd., Welwyn Garden City, Hertfordshire, UK).

#### Recording

Muscle action potentials were recorded with a concentric electromyography (EMG) needle electrode (25 mm × 30 G, Dantec DCN, Natus Manufacturing Limited, Galway, Ireland), which was positioned approximately 20 mm proximal to the stimulating needle electrode. A non-polarizable surface electrode (Red Dot, 3 M Health Care, Borken, Germany) placed on the skin at the lateral malleolus or the patella served as ground electrode. The stimulation and recording electrodes were inserted to a depth of about 7–10 mm and repositioned until a stable muscle action potential could be recorded. The electrode leads were taped to the skin to avoid displacement, especially during HUT. The position of the electrodes was checked regularly throughout testing. Signals were amplified (gain 1000, bandwidth 1.6 Hz–5 kHz) and digitized (NIDAQCARD-6062E, National Instruments Europe Corp., Debrecen, Hungary) using a sampling rate of 20 kHz. For stimulation and recording, the QtracW software (copyright Institute of Neurology, London, UK) was used. Every 2 s, test stimuli were delivered. The interval between the preceding conditioning stimulus and the test stimulus was varied in 34 steps between 1000 and 2 ms. Hence, the duration of one full MVRC recording was 3 min. The following parameters were measured from each MVRC recording: (1) MRRP, defined as the shortest interpolated interstimulus interval at which the latencies of the unconditioned and conditioned test response were identical, (2) ESN, measured as the peak percentage reduction in latency at interstimulus intervals shorter than 15 ms and (3) LSN, measured as the average percentage reduction in latency at interstimulus intervals between 50 and 150 ms. The first MVRC was recorded in the supine position before HUT, three during 10 min of HUT and two during recovery after HUT in the supine position.

### Circumference measurement and assessment of leg pain

The circumference of the right lower leg was measured 15 cm proximally to the lateral malleolus in the supine position just before HUT and at 3, 6 and 9 min of HUT. The circumference was measured with a measuring tape that was attached to the participant’s leg throughout the examination. At the same time, subjects and patients were asked to rate any leg pain they experienced on a scale from 0 to 10 (where 0 corresponds to no pain and 10 corresponds to severest pain). The rating of leg pain was explained to all participants before the beginning of the examination.

### Data analysis and statistics

MVRC data were analysed using QtracP software as described previously [8]. For statistical analyses, SPSS Statistics 25.0 (IBM Corp., Armonk, NY, USA) was used. All variables were tested for normality using the Shapiro–Wilk test. Differences in patient characteristics including autonomic function testing and age were tested using a two-tailed independent *t* test. For the analysis of MVRC data, 2 × 6 analyses of variance (ANOVA) for repeated measures with post hoc Bonferroni correction for multiple comparisons were applied. The factors consisted of (i) group (control subjects; POTS patients) and (ii) position (MVRC supine; MVRC HUT min 3; MVRC HUT min 6; MVRC HUT min 9; MVRC recovery min 3; MVRC recovery min 6). For leg pain, leg circumference and heart rate, 2 × 4 repeated-measures ANOVAs with post hoc Bonferroni correction for multiple comparisons were applied. The factors consisted of (i) group (control subjects; POTS patients) and (ii) position (MVRC supine; HUT min 3; HUT min 6; HUT min 9). Pearson correlations were calculated for relative values of MVRC parameters and relative values of leg circumference and leg pain (differences between the baseline supine and 9-min HUT position). Data are reported as mean ± standard deviation (SD). A *P* value < 0.05 was defined as statistically significant.

## Results

Table 1 summarizes participant characteristics and shows the results of autonomic function testing. Both groups were matched regarding age and sex, and had normal cardiac parasympathetic function and normal vascular sympathetic innervation to the upper limbs.

**Table 1:** Participant characteristics and autonomic function testing

	Control (*N* = 10)	POTS (*N* = 10)	*P* value
Age (mean [range])	27.2 [23–35]	24.6 [19–31]	0.126
Sex female (%)	100	100	
Heart variability (bpm)	22.11 (± 4.05)	23.75 (± 6.78)	0.558
Valsalva ratio	1.84 (± 0.38)	1.93 (± 0.55)	0.712
Cold pressor ∆SBP (mmHg)	19.56 (± 6.57)	23.8 (± 9.02)	0.287
Cold pressor ∆DBP (mmHg)	15.22 (± 7.28)	18.6 (± 9.52)	0.426
Cold pressor ∆HR (bpm)	7.33 (± 7.44)	6.00 (± 12.25)	0.792

Values are presented as mean (± standard deviation) if not differently indicated. Age is given in years. Heart rate variability was calculated as the mean difference in heart rate between the end of inspiration and the end of expiration during six respiratory cycles at a frequency of 0.1 Hz. **∆** values refer to the difference in corresponding values measured before and after 60 s of application of a cold pack to the left wrist

*POTS* postural tachycardia syndrome; *bpm* beats per minute; *SBP* systolic blood pressure; *DBP* diastolic blood pressure; *HR* heart rate

**Fig. 2:**
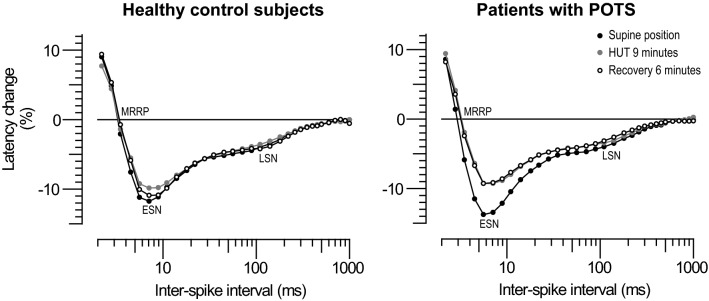
Illustration of mean muscle velocity recovery cycles. Mean muscle velocity recovery cycles with percentage changes in latency plotted as a function of interstimulus interval that were measured in the supine position before head-up tilt (black filled dots), after 9 min of head-up tilt (grey filled dots) and after 6 min of recovery in the supine position after head-up tilt (black empty dots) are shown for control subjects and patients with postural tachycardia syndrome separately. *POTS* postural tachycardia syndrome; *HUT* head-up tilt; *MRRP* muscle relative refractory period; *ESN* early supernormality; *LSN* late supernormality

**Fig. 3:**
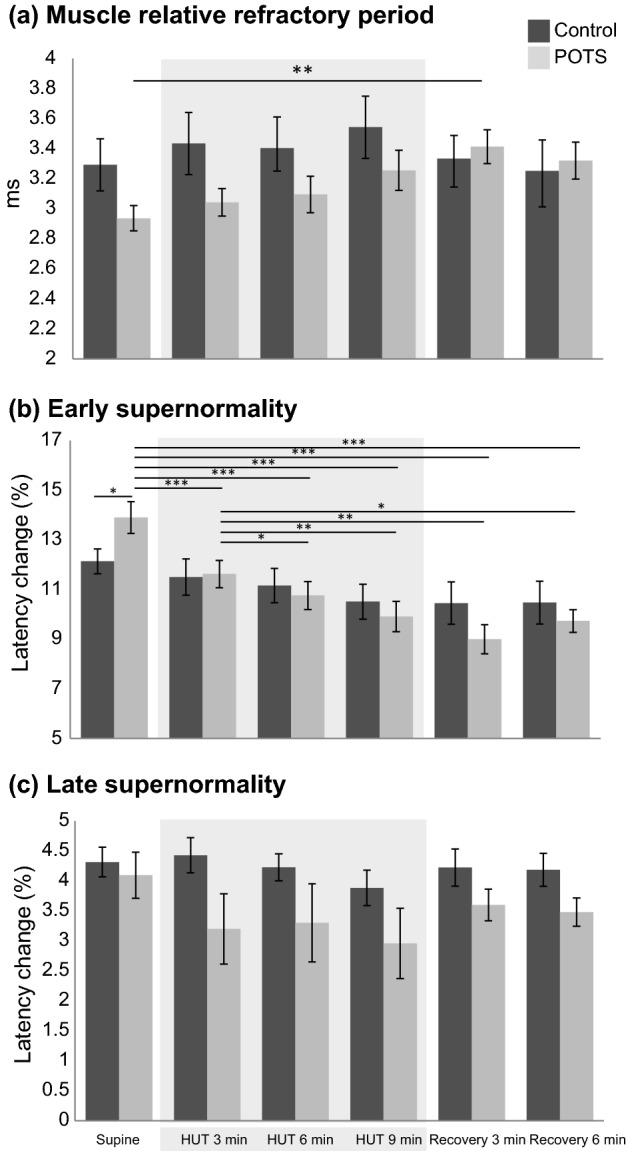
Results of post hoc comparison of muscle excitability data. **a** Bar graphs of muscle relative refractory period for healthy control subjects (dark grey) and patients with postural tachycardia syndrome (light grey) assessed at the supine position before head-up tilt, during head-up tilt at min 3, 6 and 9, and during supine recovery after head-up tilt at min 3 and 6. The same is shown in (**b**) for early supernormality and in (*c*) for late supernormality. *POTS*, postural tachycardia syndrome; *HUT*, head-up tilt. *p ≤ 0.05; **p ≤ 0.01; ***p ≤ 0.001

In all participants, six consecutive MVRCs were successfully recorded. In Fig. 2 the mean recordings measured in the supine, in the 9-min HUT position and after 6 min of recovery in the supine position are shown separately for control subjects and patients with neuropathic POTS. The results of muscle excitability testing are reported in Table 2 and illustrated in Fig. 3. The repeated-measures ANOVA for MRRP showed a significant effect for position (*F* = 4.07, *p* = 0.022) and no significant interaction. Post hoc pairwise comparisons showed a reduction in MRRP from the supine to the 3-min recovery position (*p* = 0.023) in patients only. The same analysis for ESN showed a significant effect of position (*F* = 14.39, *p* < 0.001) and a significant interaction (*F* = 3.59, *p* = 0.021). Post hoc pairwise comparisons showed an increased ESN in the baseline supine recording in patients compared to healthy subjects (*p* = 0.044). Furthermore, ESN decreased in patients from the supine to the 3-min HUT (*p* < 0.001), 6-min HUT (*p* < 0.001), 9-min HUT (*p* < 0.001), 3-min recovery (*p* < 0.001) and 6-min recovery position (*p* < 0.001). Additionally, from the 3-min HUT, ESN continued to decrease to the 6-min HUT (*p* = 0.015), 9-min HUT (*p* = 0.002), 3-min recovery (*p* = 0.002) and 6-min recovery position (*p* = 0.012) in patients only. In the control group no significant changes were found. The analysis for LSN did not reveal a significant effect for position or a significant interaction, but showed a significant effect for group (*p* = 0.047). Post hoc pairwise comparisons did not show any significant differences between or within the two groups.

**Table 2:** Results of muscle excitability testing and head-up tilt examination

		Supine	HUT min 3	HUT min 6	HUT min 9	Recovery min 3	Recovery min 6
MRRP (ms)	POTS	2.94 (± 0.27)	3.02 (± 0.29)	3.09 (± 0.38)	3.26 (± 0.42)	3.41 (± 0.11)	3.32 (± 0.12)
	Control	3.28 (± 0.58)	3.43 (± 0.69)	3.39 (± 0.51)	3.54 (± 0.62)	3.33 (± 0.19)	3.25 (± 0.24)
ESN (%)	POTS	13.9 (± 2.03)	11.62 (± 1.75)	10.76 (± 1.77)	9.91 (± 1.94)	8.99 (± 0.59)	9.74 (± 0.46)
	Control	12.13 (± 1.58)	11.50 (± 2.32)	11.16 (± 2.18)	10.51 (± 2.23)	10.46 (± 0.86)	10.47 (± 0.86)
LSN (%)	POTS	4.09 (± 1.21)	3.20 (± 1.85)	3.30 (± 2.06)	2.96 (± 1.86)	3.59 (± 0.27)	3.48 (± 0.24)
	Control	4.31 (± 0.78)	4.43 (± 0.93)	4.23 (± 0.71)	3.88 (± 0.93)	4.22 (± 0.31)	4.18 (± 0.27)
Circumference (cm)	POTS	25.38 (± 2.13)	25.51 (± 2.13)	25.61 (± 2.16)	25.68 (± 2.12)		
	Control	27.83 (± 3.28)	27.88 (± 3.24)	27.94 (± 3.21)	27.94 (± 3.21)		
Pain	POTS	0 (± 0)	0 (± 0)	3.1 (± 2.69)	3.8 (± 3.39)		
	Control	0 (± 0)	0 (± 0)	0.3 (± 0.67)	0.5 (± 0.85)		
Heart rate (bpm)	POTS	83 (± 12.20)	103 (± 18.27)	107 (± 17.78)	111 (± 18.56)		
	Control	68 (± 7.79)	86 (± 13.62)	80 (± 8.98)	84 (± 8.88)		
Systolic blood pressure (mmHg)	POTS	116.00 (± 1.31)	123.11 (± 3.54)	121.22 (± 3.63)	120.67 (± 3.53)		
	Control	119.30 (± 4.07)	124.70 (± 3.99)	125.60 (± 4.25)	121.20 (± 4.02)		
Diastolic blood pressure (mmHg)	POTS	60.56 (2.01)	72.44 (± 2.96)	65.78 (± 2.75)	65.22 (± 4.3)		
	Control	64.30 (± 2.78)	73.40 (± 3.09)	71.10 (± 3.81)	68.30 (± 4.36)		

Mean (± standard deviation) of muscle velocity recovery parameters, measured in the supine position before head-up tilt (HUT), during 10 min of HUT and for 6 min of supine recovery after HUT, as well as mean (± standard deviation) circumference of the lower leg, leg pain level (scale 0–10) and heart rate, assessed in the supine position before HUT and during 10 min of HUT are shown. ESN and LSN are given as latency change (%)

*HUT* head-up tilt; *POTS* postural tachycardia syndrome; *MRRP* muscle relative refractory period; *ESN* early supernormality; *LSN* late supernormality; *bpm* beats per minute

Regarding the biological parameters, the repeated-measures ANOVA for circumference of the lower leg showed a significant effect for position (*F* = 7.11, *p* = 0.005). Pairwise comparisons showed that in patients, the circumference increased from the supine to the 6-min (*p* = 0.011) and the 9-min HUT position (*p* = 0.001). Looking at the HUT phase only, the circumference also increased from the 3-min to the 9-min HUT position (*p* = 0.004) and from the 6-min to the 9-min HUT position (*p* = 0.024). No such changes were found for the control group. The analysis for leg pain revealed a significant effect for position (*F* = 7.45, *p* = 0.005) and group (*p* = 0.006), as well as a significant interaction (*F* = 4.84, *p* = 0.022). Pairwise comparisons showed that at 6 and 9 min of HUT, the pain levels were higher in patients than in healthy subjects (*p* = 0.005 and *p* = 0.008, respectively). Furthermore, the pain levels significantly increased in patients from the supine to the 6-min and 9-min HUT position, and from the 3-min to the 6-min and 10-min HUT position (all *p* = 0.001). Again, no such changes were found for the control group. The decrease in ESN from the supine to the 9-min HUT position correlated negatively with the increase in leg pain during the same time period (*r* =  −0.613, *p* = 0.004). All other correlations between MVRC parameters and leg circumference and pain were not significant. During HUT, patients experienced a mean heart rate increase of 28 bpm, and healthy subjects an increase of 15 bpm (*p* < 0.001).

## Discussion

In this study, muscle excitability measurements were used to examine muscle membrane properties depending on body position in patients with neuropathic POTS and healthy subjects in order to investigate the aetiology of muscle pain in neuropathic POTS. Compared to control subjects, patients with neuropathic POTS showed altered muscle excitability in the baseline examination while supine, as well as progressive changes during and a delayed recovery after HUT. In parallel, a position-dependent increase in lower leg circumference and in leg pain levels was found.

Looking at the baseline MVRC recorded in the supine position before HUT, ESN was increased in neuropathic POTS patients compared to healthy subjects. Similarly, there was a tendency of shorter MRRP in neuropathic POTS. ESN has been shown to be a highly sensitive parameter of membrane potential [8, 9]. The increase in ESN together with the shorter MRRP is an indicator of muscle membrane hyperpolarization. A previous study evaluating muscle excitability measurements in the brachioradialis muscle of healthy subjects before and after a 2-week force training showed that ESN increased from 12.0 to 13.6% and MRRP decreased from 3.2 to 2.9 ms [24]. The authors interpreted these changes as an expression of a hyperpolarization of the muscle membrane due to increased Na^+^/K^+^ pump density induced by the force training. The increased Na^+^/K^+^ pump density itself leads to heightened pump efficacy, which in turn hyperpolarizes the muscle membrane [25]. The ESN and MRRP values measured in patients with neuropathic POTS in the supine position before HUT (ESN 13.9%; MRRP 2.94 ms) correspond to the values measured in healthy subjects with hyperpolarized muscles after force training. One possible interpretation is that the muscles of patients with neuropathic POTS hyperpolarize as a consequence of the orthostatic stress, as in the upright body position muscle activity may be constantly increased due to the altered blood perfusion (see below). The excessive blood pooling during standing may cause an “ischemia-like” environment as it exists also during forced muscle activity, e.g., during training. As a consequence, the density of the Na^+^/K^+^ pump increases, resulting in membrane hyperpolarization at rest.

Furthermore, muscle excitability measurements showed progressive changes in MVRC parameters during HUT and delayed recovery in patients with neuropathic POTS, but remained stable in controls. ESN decreased significantly during HUT and continued to decrease during the first 3 min of the recovery phase, not reaching the baseline level by the end of the observation period of 6 min. In parallel, MRRP increased during HUT and continued to increase during the first minutes of supine recovery. This pattern of decreasing ESN and prolonged MRRP has been shown to be a sign of muscle membrane depolarization. Similar changes have been previously reported in a study investigating muscle excitability in healthy subjects during induced muscle ischemia [8] and have been observed in the trapezius muscle of patients with orthostatic hypotension who experience coat-hanger ache upon standing [13]. LSN was significantly lower overall in patients with neuropathic POTS compared to healthy subjects, but did not change as a function of body position in either group. While both ESN and MRRP are closely related to muscle membrane potential, LSN is thought to be a parameter representing transverse tubular function. Our results therefore indicate an altered transverse tubular state in neuropathic POTS patients that is independent of body position. In a recent study, we investigated changes in MVRC parameters during muscle fatigue in healthy subjects, which was induced by intermittent 37 Hz stimulation. This stimulation paradigm produced a gradual decrease in LSN, with delayed recovery after cessation of stimulation, suggesting that longer muscle activation may lead to a progressive decrease in LSN [26]. The mechanisms that are reflected by LSN are assumed to depend on ion accumulation in the transverse tubule system and passive diffusion of accumulated ions during the recovery phase [8, 27–29]. Earlier studies have shown that chloride channels and thus chloride conductance in the transverse tubular system counteract the fatiguing effects of potassium accumulation to maintain muscle excitability [29, 30].

In patients with neuropathic POTS, a continuous increase in the circumference of the lower leg was measured during the HUT position, most likely due to the increased pooling of blood as a consequence of the impaired vasoconstriction caused by the peripheral sympathetic dysfunction [2, 4, 31]. The occurrence of increased leg circumference and blood volume in patients with neuropathic POTS during HUT has also been shown in previous studies [32, 33]. Furthermore, Kadamati et al. (2018) found that during HUT, deoxygenated blood accumulates in the legs of patients with POTS and that the recovery of muscle oxygenation after HUT is delayed compared to healthy controls [33]. Thus, it is possible that in patients with neuropathic POTS, the lack of oxygenated blood may lead to an ischemia-like state while standing, which could be the source of muscle pain and exercise intolerance, and induces hyperpolarization in the supine position. This hypothesis is supported by the finding that patients reported an increase in leg pain during HUT, which also correlated with the reduction of ESN. Additionally, the findings of both Kadamati and colleagues (2018) and the present study indicate that in contrast to other orthostatic symptoms in neuropathic POTS such as light-headedness, palpitations and nausea, the inadequate perfusion of the lower extremities and the reduced muscle excitability do not normalize immediately in the supine position. This is consistent with patient reports describing delayed recovery of leg pain.

Taken together, the findings revealed that patients with neuropathic POTS during HUT show a decreasing ESN and a prolongation of MRRP as an indicator of progressive muscle membrane depolarization, probably caused by the ischemia-like state. This altered metabolic condition not only affects muscle activity during standing, and as a consequence delays muscle recovery, but also leads to non-orthostatic changes in muscle membrane properties, which are reflected in the increased ESN at rest as a sign of muscle membrane hyperpolarization as well as in an overall decrease in LSN, indicating changed transverse tubular function. Patients with neuropathic POTS are known to experience exercise intolerance [2]. The overall decreased LSN as well as the progressive muscle membrane depolarization during HUT explain the reduced potential for muscle activation and the occurrence of exercise intolerance. The occurrence of muscle fatigue depends on the degree of membrane depolarization. Thus, a more hyperpolarized muscle membrane potential at rest will delay the onset of critical depolarization for fatigue. These findings taken together support regular exercise as a treatment modality in neuropathic POTS [34] and at least partially explain its beneficial effect: Training increases hyperpolarization in the long term and reduces muscle membrane depolarization. Furthermore, measures that reduce pooling and enhance perfusion of the lower extremities can be expected to be beneficial in neuropathic POTS.

### Limitations

The results of the present study should be viewed in the light of some limitations. The study was conducted with a limited sample size; thus, the reported findings should be confirmed in a larger patient collective. Furthermore, we did not examine patients with neuropathic POTS who were not experiencing orthostatic leg pain. Due to the rare occurrence and the consequently extremely small sample size, we could not include patients with neuropathic POTS without leg pain as a further control group. Therefore, we cannot say with certainty whether the changes in muscle excitability found are specific to patients with neuropathic POTS experiencing leg pain, or a feature of neuropathic POTS itself. During HUT, patients did not reach a heart rate increase of more than 30 bpm and therefore do not formally fulfil the diagnostic criteria for POTS. However, patients were allowed to eat and drink before the study and took their medications as usual with the exception of midodrine, which explains why the heart rate increase remained below 30 bpm. The only other medication besides midodrine that could have had an effect on the muscle excitability measurements is pyridostigmine. Theoretically, pyridostigmine would be associated with muscle membrane depolarization. This was not the case in our study, as the baseline muscle excitability measurements in the supine position showed the opposite (hyperpolarization). Furthermore, the aim of this study was to investigate muscle membrane properties depending on the patient’s body position, and therefore we were primarily interested in the within-subject changes occurring during HUT. Within-subject differences could not be influenced by medication; such an influence would only be possible for between-subject differences. For these reasons we conclude that a possible confounding effect of medication is unlikely and, most importantly, the main research question could be investigated unaffected by the intake of the listed medications. Finally, as patients experience a delayed recovery of muscle excitability parameters after being upright, and the baseline MVRC measurement in the supine position was recorded after a 20- to 30-min supine rest period, we cannot fully exclude the possibility that this measurement may have been affected by too short a rest period before the start of the recordings.

## Conclusion

In summary, our results show that in patients with neuropathic POTS, muscle fibres are hyperpolarized in the supine position and depolarize progressively during HUT with delayed recovery. Depolarization of muscle fibres during HUT was paralleled by an increase in the circumference of the lower leg and leg pain levels. Hence, the altered perfusion as a consequence of blood stasis may cause an ischemia-like state of the muscles, which could explain the occurrence of orthostatic leg pain in patients with neuropathic POTS.
